# Diagnostic Risk Prediction Models for Upper Gastrointestinal Cancers: A Systematic Review

**DOI:** 10.1158/1055-9965.EPI-24-1714

**Published:** 2025-05-22

**Authors:** Tyler Seyhan Saunders, Pawandeep Virpal, Maria Andreou, Asha Parmar, Christina Derksen, Oleg Blyuss, Fiona M. Walter, Garth Funston

**Affiliations:** Centre for Cancer Screening, Prevention and Early Diagnosis, Wolfson Institute of Population Health, Queen Mary University of London, London, United Kingdom.

## Abstract

Upper gastrointestinal (UGI) cancers are often detected late. Risk prediction models could facilitate earlier detection by identifying patients at risk for further investigation. We systematically reviewed evidence on UGI diagnostic risk prediction models. A search of MEDLINE, Embase, and CENTRAL was conducted for studies reporting on the development and/or validation of diagnostic risk prediction models for UGI cancers (pancreatic, gastric, esophageal, gallbladder, and/or biliary tract). Studies had to report at least one quantitative measure of model performance to be eligible for inclusion. A total of 82 studies describing 162 UGI risk models were included. Models predicted gallbladder (*n* = 6), gastric (*n* = 25), esophageal (*n* = 34), gastroesophageal (*n* = 14), and pancreatic (*n* = 83) cancers. Most models used logistic regression, but machine learning was increasingly used from 2019. In total, 366 unique variables were incorporated across models. Only 33 models were externally validated, with 15 achieving an AUC ≥0.80. This review highlights that several models perform well in predicting UGI cancers on external validation. Future research is needed to compare the best-performing models and assess their clinical utility, acceptability, and cost-effectiveness. Given the significant overlap in at-risk populations and predictors across UGI cancers, there may also be scope to develop UGI “multicancer” models.

## Introduction

Upper gastrointestinal (UGI) cancers, including pancreatic, gastric, esophageal, gallbladder, and biliary tract cancers, remain significant contributors to global cancer mortality, with poor survival outcomes worldwide (1). Pancreatic cancer, for example, has one of the lowest survival rates of any cancer, with a five-year net survival rate of less than 10% in England (2) and a five-year relative survival rate of 13% in the United States (3). Gastric, esophageal, gallbladder, and biliary tract cancers all have five-year survival rates of 36% or less in England (2, 4, 5) and the United States (6–8). Late-stage diagnosis is a major factor contributing to poor outcomes. For example, localized pancreatic cancer has a three-year net survival of over 25% in England, compared with 15% for regional and 1% for distant pancreatic cancer (9). However, most pancreatic cancers are diagnosed at a distant stage (9).

Some national guidelines advocate screening for pancreatic cancer in higher-risk groups (10, 11), whereas several countries with high incidence rates offer screening programs for esophageal and gastric cancer (12, 13). But, in most countries, screening programs for asymptomatic individuals at average risk of UGI cancers are not recommended. Most patients with UGI cancer are diagnosed after they develop symptoms and present to their doctor. However, most symptoms of UGI cancers, such as weight loss, abdominal pain, and fatigue (14, 15), are nonspecific and easily misattributed to benign conditions by both patients and clinicians (16, 17). This poses a diagnostic challenge. Earlier diagnosis of UGI cancers, either through asymptomatic screening or earlier identification of cancer in patients with nonspecific symptoms, could improve outcomes by allowing for timely intervention, expanding treatment options, and potentially improving long-term survival and enhancing quality of life (18).

To address the challenges of late diagnosis, various risk prediction models have been developed to quantify an individual’s risk of UGI cancer and guide both surveillance and testing decisions. These models incorporate a range of established risk factors—including demographics, symptoms, novel biomarkers, routine lab tests, imaging, medications, and genetic data—and are intended to accurately quantify cancer risk and identify patients for further investigation. Increasingly, models are being integrated into the clinical decision-making processes. QCancer and electronic risk assessment tools (eRATs) are available to support decisions on further investigations in symptomatic individuals in UK clinical practice (19, 20), whereas new algorithms, such as the Enriching New-Onset Diabetes for Pancreatic Cancer (ENDPAC) model (21), are being used in clinical screening trials (22). Accurate prediction tools for UGI cancer could aid risk stratification, helping to select appropriate patients for further investigation based on individualized risk, avoiding potentially unnecessary tests, referrals, and harm while facilitating more timely investigation in patients with cancer. However, the utility of risk prediction models in identifying undiagnosed UGI cancers hinges on their accuracy and applicability in real-world settings. Additionally, given the overlap in risk factors, symptoms, and shared diagnostic pathways for the major UGI cancers, there is a rationale for examining these models collectively rather than in isolation as previous systematic reviews have (23–26). Recent evidence indicates that although the risk of individual cancers in those with symptoms or abnormal tests may be low, the combined risk of cancers may be above national thresholds for further investigation (27–30). Furthermore, multicancer early detection (MCED) tests (31) offer a promising approach in identifying multiple cancer types, particularly as reported performance in UGI cancers is high, which could enhance detection in patients presenting with nonspecific symptoms.

This review aims to identify existing diagnostic prediction models for each UGI cancer (and combinations of UGI cancers), to summarize how these models were developed and the predictors included across cancer types, and to critically examine model performance.

## Materials and Methods

This review was prospectively registered on PROSPERO (CRD42024511177) and is reported according to the Preferred Reporting Items for Systematic Reviews and Meta-Analyses (PRISMA) guidelines (32).

### Search strategy

An electronic literature search of MEDLINE, Embase, and CENTRAL was performed from January 1, 2000, to October 2023 for primary studies. The search strategy combined keywords and Medical Subject Headings (MeSH) related to UGI cancers, risk prediction models, and statistical/machine learning (ML) methods (full search terms can be found in Supplementary Table S1). Reference lists of studies and reviews were manually searched to identify additional suitable studies. No language restrictions were applied. Only studies published in peer-reviewed journals were eligible for inclusion, and conference abstracts were excluded.

### Eligibility criteria

Studies that fulfilled the following inclusion criteria were included: (i) the study described the development and/or validation of a multivariate diagnostic prediction model designed to identify patients with a UGI cancer, (ii) the study investigated any UGI cancer alone or in combination, (iii) the study reported at least one quantitative measure of model performance (discrimination, calibration, or threshold accuracy metrics), and (iv) the majority of the sample comprised individuals above 18 years old. For the purpose of this review, a UGI cancer was defined as one of the following: pancreatic, esophageal, gallbladder, gastric, or extrahepatic biliary tract cancer. Studies were excluded if (i) the study focused solely on prognostic models or models aiming to provide an indication of relapse or recurrence; (ii) the reported model did not include a patient-related variable such as demographics, symptoms, or risk factors (to exclude models that were focused exclusively on multipanel biomarker tests); and (iii) the study was a nonprimary study, not peer-reviewed, or was a conference abstract. All study designs and recruitment settings were eligible for inclusion.

Independent researchers (T.S. Saunders, M. Andreou, A. Parmar) screened studies for inclusion/exclusion and resolved any discrepancies through discussion.

### Data extraction

Data were extracted from eligible studies using Covidence software by pairs of independent researchers (T.S. Saunders, M. Andreou, A. Parmar, P. Virpal). This included study characteristics (year, country, design, setting), sample characteristics (recruitment, number of participants, sex, ethnicity, age, UGI cancer type), model characteristics (candidate predictors, predictors included in the final model, development method, missing data), and model performance measures (discrimination measure, any validation methods, calibration metrics, performance by subgroups: age/sex/ethnicity/geographical location/socioeconomic status). The primary outcome was a UGI cancer of interest. Data were also extracted where a study reported on model performance differences between different population groups such as ethnicities, socioeconomic status, and sex, to examine whether models may exacerbate any existing health disparities between population groups. Data extraction was conducted using an extraction template, which was piloted before use. Where a study described multiple models, data for each were extracted separately. Any discrepancies were resolved through discussion.

### TRIPOD classification

Studies were classified according to the transparent reporting of a multivariable prediction model for individual prognosis or diagnosis (TRIPOD) criteria (33). The criteria were developed with the aim of improving the transparency of the reporting of risk prediction model studies and provide a useful framework for categorizing risk prediction model studies. Studies can be classified into the following categories: 1a (development only), 1b (development and validation using resampling), 2a (random split-sample development and validation), 2b (nonrandom split-sample development and validation), 3 (development and validation using separate data), and 4 (validation only).

### Risk of bias

The Quality Assessment of Diagnostic Accuracy Studies 2 (QUADAS-2; ref. 34) tool was used to evaluate the risk of bias and applicability of included studies. The QUADAS-2 tool consists of four key domains: patient selection, index test, reference standard, and flow and timing. Each domain is assessed in terms of risk of bias, receiving a rating of “high,” “low,” or “unclear” (where insufficient information is provided to make a judgment). Signaling questions are included to assist in judgments about risk of bias. The first three domains also receive a rating of “high,” “low,” or “unclear” for concerns regarding applicability. The risk of bias was assessed independently by two reviewers using the QUADAS-2 tool. Any discrepancies were resolved by discussion.

### Narrative synthesis

Model performance metrics were selected as the outcome measure of the review, with AUC chosen as the primary measure as previous reviews have identified it as the most commonly reported outcome (23, 24, 26). If AUC was not reported, sensitivity and specificity were chosen instead. Meta-analysis was not possible due to heterogeneity in terms of study design, study setting, model development methods, and sample characteristics. Therefore, studies were grouped by study setting and UGI cancer. This grouping was necessary as the characteristics of samples recruited from different settings may influence performance, with clinical samples more likely to report symptoms than population-based samples.

There was variation in the categorization of gastroesophageal cancers, with eight studies including it as a single category, whereas others treated esophageal cancer and gastric cancer as distinct entities. This inconsistency in categorization could impact the comparability of models and outcomes across studies, and so gastroesophageal was treated as a separate UGI category in the current review. Performance metrics were compared in tabular and graphical form and summarized with a narrative synthesis. Externally validated models (per TRIPOD criteria) were emphasized in the analysis. Models were considered unique if they included different variables, employed different statistical methods, or aimed to detect different stages of UGI cancer. If an existing model was updated with new variables, this was classified as a unique model. Model development methods were grouped into either “ML” or “conventional statistical” methods. For the purpose of this review, any method more advanced than logistic regression was classified as “ML,” whereas logistic regression itself was classified as a conventional statistical method.

### Data availability

Data sharing is not applicable to this article as no data were created in this systematic review.

## Results

### Search results

**Figure 1.**
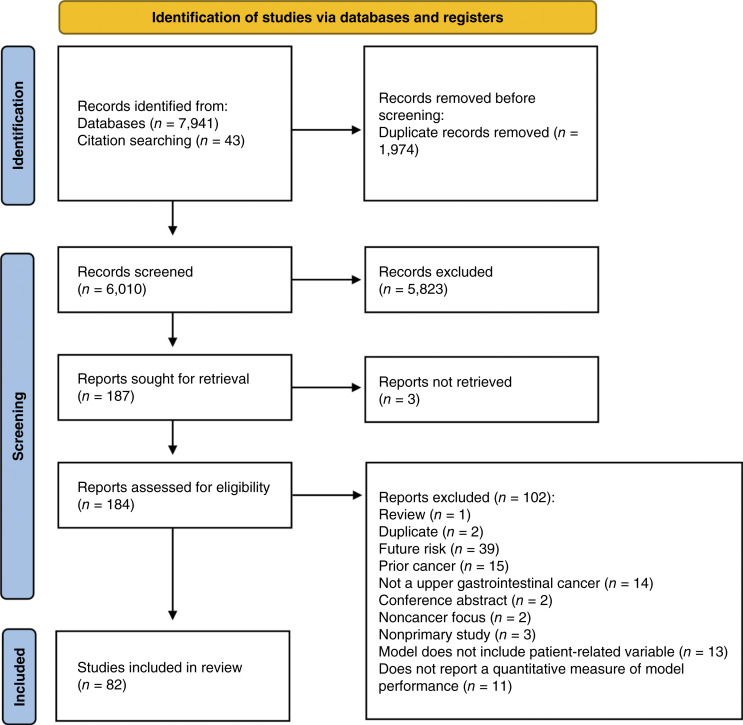
Flow diagram of search strategy. Diagram showing the number of titles/abstracts screened, full texts reviewed, and studies included.

After the initial search, 7,941 studies were identified for screening. A further 43 studies were identified from manual reference list searching. After the removal of duplicates, titles and abstracts of 6,010 studies were screened. After full-text screening of 187 studies, 82 studies met the inclusion criteria (see Fig. 1).

### Study characteristics

Eighty-two studies included in the current review (see Supplementary Table S2) focused on gallbladder (*n* = 6), gastric (*n* = 18), esophageal (*n* = 14), gastroesophageal (*n* = 8), and pancreatic (*n* = 33) cancers—with three studies focusing on both pancreatic and gastroesophageal cancers. Most studies were conducted in China (*n* = 35), followed by the United Kingdom (*n* = 11) and the United States (*n* = 9).

A total of 162 unique models were described: six for gallbladder, 25 for gastric, 34 for esophageal, 14 for gastroesophageal, and 83 for pancreatic cancer. Only one model targeted multiple UGI cancers simultaneously (esophageal, pancreatic, and gastric cancers; ref. 35), and none focused on extrahepatic biliary cancer.

According to TRIPOD criteria, most studies were classified as 2a (random split-sample development and validation, *n* = 24, 29.3%), followed by 1b (development and validation using resampling, *n* = 20, 23.2%) and 1a (development only, *n* = 18, 22%). Other classifications included 3 (development and validation using separate data, *n* = 14, 17.1%), 4 (validation only, *n* = 5, 7.3%), and 2b (nonrandom split-sample development and validation, *n* = 1, 1.2%).

Study designs predominantly included case–control (*n* = 49) and cohort (*n* = 33) designs. Most were conducted in hospital or clinic settings (*n* = 61), with others in population-level settings (*n* = 13) and primary care (*n* = 5). Data sources varied, with most studies using existing health records (*n* = 39), followed by in-person interviews (*n* = 11) and questionnaires (*n* = 9).

### Risk prediction model development

**Figure 2.**
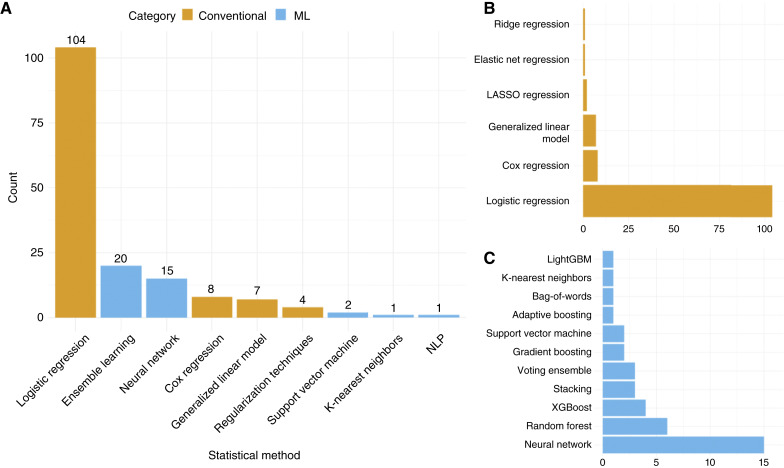
Statistical methods used to develop each model. **A,** Bar chart showing the number of models developed with different methods, either ML (blue) or conventional statistics (orange) **B,** The most common methods used for conventional statistics and the number of models. **C,** The most common methods used for ML techniques and the number of models.

Model development methods varied across studies. Of all included models, 123 were developed using conventional statistical methods (Fig. 2A), most commonly logistic regression (*n* = 104), followed by Cox regression (*n* = 8) and logistic generalized linear models (*n* = 7; Fig. 2B). Thirty-nine models were developed using ML, such as ensemble learning techniques (*n* = 20) and neural networks (*n* = 15; Fig. 2C). Consistent with expectations, the use of ML increased over time, with the first ML models appearing in 2019, compared with conventional statistical models, which have been published since at least 2005.

### Missing data

Of all included studies, only 17 studies reported the proportion of missing data for variables of interest (21, 36–51) although 31 studies reported how missing data were handled. Complete-case analysis was used in 17 studies (39–42, 44, 46, 48, 50, 52–58), in which patients were excluded if they had missing data for variables of interest. The remaining studies reported that missing values were imputed using multiple imputation (19, 20, 35, 37, 38, 43, 45, 47, 49, 59–62).

### Risk prediction model variables

A total of 366 unique variables were included across models, grouped into 12 categories: biomarkers (including novel biomarkers), comorbidities, demographics, family history, genetics (e.g., SNPs, polygenic risk scores), general health (e.g., perceived health status), imaging, standard lab tests (e.g., platelet counts), lifestyle (e.g., smoking, alcohol, and diet), medications, symptoms, and other variables (e.g., cytological variables). Supplementary Table S3 lists all unique variables in each category, while Supplementary Table S4 provides details on variable selection and cutoffs/categorizations of variables in the 15 best-performing externally validated models.

**Figure 3.**
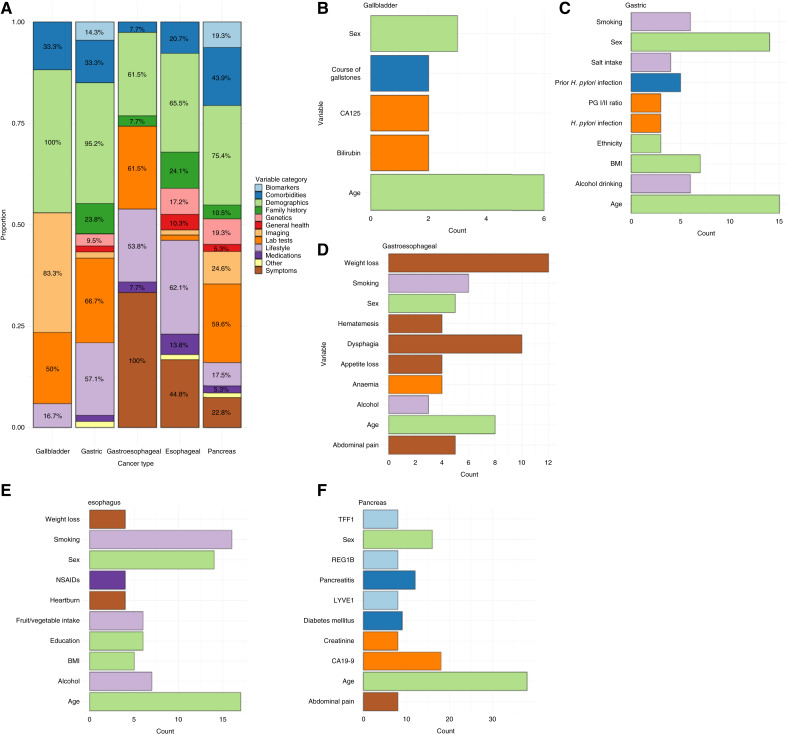
Variables included in models across UGI cancer types. **A,** Stacked bar chart showing the proportion of models containing variables of each category by UGI cancer type. **B–F,** Up to the top 10 most common variables included in models for each cancer type: (**B**) gallbladder, (**C**) gastric, (**D**) gastroesophageal, (**E**) esophageal, and (**F**) pancreatic. Data shown are the number of models containing each variable.

Figure 3A–F illustrate the proportion of variable categories by UGI cancer type. Several variables were shared across models for different UGI cancers. Notably, three variables—age, sex, and alcohol consumption—are included in at least one model for each UGI cancer. Ethnicity, smoking, and body mass index (BMI) are present in models for all UGI cancers except gallbladder cancer. Additionally, CA125 and carcinoembryonic antigen (CEA) are included in at least one model for all UGI cancers except for gastroesophageal models. Conversely, some variable categories differed by cancer type; for example, imaging variables were more common in gallbladder models, whereas symptom variables were prevalent in gastroesophageal models but absent from gallbladder and gastric models.

**Figure 4.**
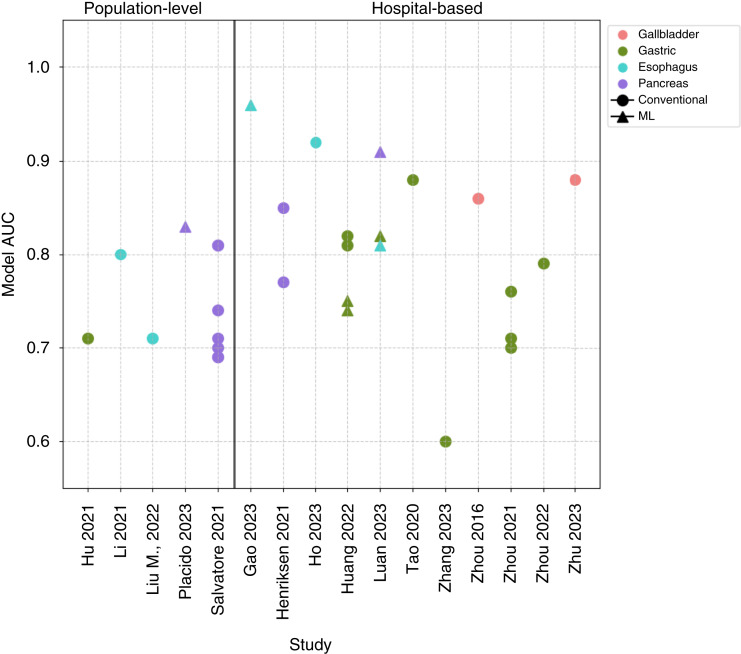
AUCs reported in external validation studies. Dot plot showing the AUC reported by each external validation study, separated by study setting. Each data point represents a single model. Data points are colored according to UGI cancer site (red, gallbladder; green, gastric; blue, esophageal; purple, pancreatic). Data point shaped corresponds to the model development method (circle, conventional statistics; triangle, ML).

### Validation

Of the 162 risk prediction models identified, 38 were described without any validation, 91 models had been internally validated, and 33 had been externally validated.

A random split-sample validation was the most commonly employed internal validation technique, in which a model was developed on a subset of the total sample and tested on the remaining sample (*n*_*models*_ = 44; refs. 19, 20, 46–48, 51, 55, 57, 59, 60, 62–70). Cross-validation was the second most common internal validation method (*n*_*models*_ = 36; refs. 40, 41, 43–45, 49, 61, 71–81), followed by bootstrapping (*n*_*models*_ = 17; refs. 36, 53, 82–88).

Nineteen studies reported the external validation of 33 different models (see Table 1). Thirteen studies reported the development of a prediction model and its evaluation on separate data (35, 37, 58, 61, 73, 88–95), whereas six studies evaluated the performance of an existing prediction model on separate data (50, 52, 54, 96–98). Of these studies, five were conducted at the population level, and 14 were conducted in a hospital/clinic setting.

**Table 1.** Table of external validation studies.

Lead author (year)	Outcome	Model development	Variable categories included in the model										AUC (95% CI)
			D	C	Sx	Img	Lab	LS	Gen	GH	FH	Oth	
Population-based													
Hu (2021)	GC	LR	●				●	●					0.71 (0.64–0.77)
Li (2021)	OSCC	LR	●	●	●			●			●		0.80 (0.75–0.84)
Liu. M (2022)	OSCC	LR	●					●			●		0.71 (0.65–0.78)
Placido (2023)	PC	Neural network		●	●		●						0.83 (0.83–0.83)^a^
Salvatore (2021)	PC	GLM	●						●				0.69 (0.67–0.71)
Salvatore (2021)	PC	GLM	●					●	●				0.69 (0.69–0.71)
Salvatore (2021)	PC	GLM	●					●	●				0.71 (0.69–0.73)
Salvatore (2021)	PC	GLM		●									0.70 (0.69–0.73)
Salvatore (2021)	PC	GLM	●	●				●	●				0.81 (0.79–0.82)
Salvatore (2021)	PC	GLM		●					●				0.74 (0.72–0.77)
Salvatore (2021)	PC	GLM	●	●				●	●				0.81 (0.79–0.83)
Hospital-based													
Gao (2023)	OSCC (78%) OAC (22%)	Light-gradient boosting	●	●				●		●	●	●	0.96 (0.92–0.99)
Henriksen (2021)	PDAC	LR	●						●				0.77 (0.69–0.84)
Henriksen (2021)	PDAC	LR	●				●		●				0.85 (0.79–0.91)
Ho (2023)	OSCC (7%) OAC (93%)	LR	●	●	●			●					0.92 (0.88–0.96)
Huang (2022)	GC (non-cardia)	Random forest	●	●			●						0.74 (NR)
Huang (2022)	GC (non-cardia)	K-nearest neighbors	●	●			●						0.75 (NR)
Huang (2022)	GC (non-cardia)	LASSO regression	●	●			●	●					0.82 (NR)
Huang (2022)	GC (non-cardia)	LR	●	●			●	●					0.81 (NR)
Kapoor (2005)	G-O	LR			●		●						NR
Luan (2023)	GC	Random forest	●				●						0.82 (NR)
Luan (2023)	PC	Random forest	●				●						0.91 (NR)
Luan (2023)	OC	Random forest	●				●						0.81 (NR)
Shen (2013)	MPCN	LR				●	●						NR
Tao (2020)	GC	LR	●	●			●	●					0.88 (0.85–0.92)
Yokoyama (2013)	OSCC (95%) OAC (5%)	LR						●					NR
Zhang (2023)	GC	LR	●				●	●			●		0.60 (NR)
Zhou (2016)	GBC	LR	●	●		●		●					0.86 (NR)
Zhou (2021)	GC	LR	●					●			●		0.76 (0.71–0.82)
Zhou (2021)	GC	LR	●					●			●		0.71 (0.61–0.81)
Zhou (2021)	GC	LR	●					●			●		0.70 (0.57–0.83)
Zhou (2022)	GC	LR	●				●						0.79 (NR)
Zhu (2023)	GBC	LR		●		●	●						0.88 (0.82–0.94)

Abbreviations: C, comorbidities; D, demographics; DAC, pancreatic ductal adenocarcinoma;  FH, family history; GBC, gallbladder cancer; GC, gastric cancer; Gen, genetics; GH, general health; GLM, generalized linear model; G-O, gastro-oesophageal cancer; Img, imaging; Lab, lab test; LASSO, least absolute shrinkage and selection operator; LR, logistic regression; LS, lifestyle; MPCN, malignant pancreatic cystic neoplasm; NR, not reported; OAC, oesophageal adenocarcinoma; OC, oesophageal cancer unspecified; OSCC, oesophageal squamous cell carcinoma; Oth, other; PC, pancreatic cancer unspecified; Sx, symptoms.

a AUC = 0.83 (6-month prediction interval), 0.79 (12-month prediction interval), and 0.71 (36-month prediction interval).

### Risk prediction model performance

Model discrimination, as measured by AUC, was reported for 152 models, with 134 (88.2%) reporting moderate-to-strong performance. Of the 33 externally validated models, 30 reported AUCs, and 15 demonstrated moderate-to-strong performance.

#### Performance across statistical categories

Among the externally validated models, 26 were developed with conventional statistics, and seven were developed using ML. ML models reported a slightly higher median AUC (0.82, IQR = 0.13) than conventional methods (AUC = 0.77, IQR = 0.11) although direct comparisons were not possible.

#### Performance of externally validated models in hospital-based settings

Twenty-two models were externally evaluated in hospital-based settings (35, 37, 51, 52, 58, 61, 88, 91–97). Below is a summary of models with the highest AUCs for each UGI cancer type (Fig. 4).

##### Gallbladder cancer

Two models achieved high performance. Zhou and colleagues (88) developed a model in patients with chronic cholecystitis, incorporating demographics, lifestyle, and imaging variables, achieving an AUC of 0.86. Another model included demographics, gallstone history, and blood tests (CEA, CA19-9), with an AUC of 0.88 [95% confidence interval (CI), 0.82–0.94; ref. 58].

##### Gastric cancer

Among 11 externally validated models (35, 50, 91, 94, 95), AUCs ranged from 0.60 to 0.88. Tao and colleagues (94) achieved the highest AUC (0.88) with a model, including demographics, history of *Helicobacter pylori*, lab tests (serum pepsinogen), and lifestyle factors (type of drinking water) in a very small cohort (*n* = 26). Another notable model by Huang and colleagues included demographics, anemia status, comorbidities, and smoking, achieving an AUC of 0.82 (91).

##### Gastroesophageal cancer

Only one model was externally validated, developed by Kapoor and colleagues (92) for patients referred to a rapid access UGI cancer service. The model, based on symptoms (e.g., dysphagia, weight loss) and age, achieved a high sensitivity (92.3%) but poor specificity (32.7%) at the selected threshold.

##### Esophageal cancer

Four externally validated models (35, 37, 61, 96) reported AUCs ranging from 0.81 to 0.9. The highest AUC (0.96) was reported by Gao and colleagues for a model developed in patients undergoing UGI endoscopy screening (37). It included demographics, lifestyle factors (e.g., smoking, pickled food), family history, and cytological features. Ho and colleagues (61) also reported a high AUC of 0.92 with a model including demographics, lifestyle factors (e.g., smoking, diet), comorbidities, and symptoms.

##### Pancreatic cancer

Four externally validated models reported AUCs ranging from 0.77 to 0.91 (35, 52, 93). Luan and colleagues (35) developed a model incorporating blood biomarkers (e.g., α-fetoprotein, CA19-9, and CEA) and demographics, achieving an AUC of 0.91. Henriksen and colleagues (52) updated an existing gene-based model (71) to include serum CA19-9, resulting in an AUC of 0.85 (95% CI, 0.79–0.91), although calibration was reported to be poor.

#### Performance of externally validated models in population-based settings

Eleven models were externally validated in population-based settings across five studies (Fig. 4; refs. 54, 73, 89, 90, 98).

##### Gastric cancer

Hu and colleagues (98) validated a model for high-risk individuals in China, incorporating lab tests (pepsinogen I/II ratio, gastrin-17), lifestyle variables (diet), and demographics, achieving an AUC of 0.71.

##### Esophageal cancer

Li and colleagues described a model in high-risk areas of China, incorporating demographics, family history, lifestyle (e.g., smoking, diet), symptoms, and comorbidities, with an AUC of 0.80 (95% CI, 0.75–0.84; ref. 54). Another model included similar variables such as demographics, family history, and diet, with an AUC of 0.71 (95% CI, 0.65–0.78; ref. 73).

##### Pancreatic cancer

Eight models were externally validated across two studies (89, 90), with AUCs ranging from 0.69 to 0.83. Placido and colleagues used a deep learning algorithm on electronic health record data and developed a model using demographics and comorbidities (specifically, International Classification of Diseases diagnostic codes) to achieve an AUC of 0.83 when predicting 6 months prior to diagnosis (95% CI, 0.83–0.83) and 0.71 (95% CI, 0.71–0.71) 36 months prior to diagnosis. Salvatore and colleagues described a model using UK Biobank data, incorporating demographics, comorbidities (International Classification of Diseases codes), lifestyle (e.g., smoking, alcohol), and a polygenic risk score for pancreatic cancer, reaching an AUC of 0.81 (95% CI, 0.79–0.82).

#### Performance differences across different population groups

Only two studies examined differences by population subgroups although neither was an external validation study. Chen and colleagues (65) reported an internally validated pancreatic cancer model that performed better in females (AUC = 0.85) than in males (AUC = 0.82) and showed slightly improved performance for Black (AUC = 0.84) over White participants (AUC = 0.83). Similarly, Rubenstein and colleagues (47) reported an internally validated gastroesophageal cancer model, which performed better in females (AUC = 0.83) than in males (AUC = 0.72). This model also performed better in Asian (AUC = 0.80) than in White (AUC = 0.75) and Black (AUC = 0.75) participants.

### Calibration

Calibration metrics were reported for 22 of the 33 externally validated models (37, 50, 52, 54, 58, 73, 88, 90, 91, 93, 94). The most common method of assessing calibration was the Hosmer–Lemeshow test (*n* = 14), followed by calibration plots (*n* = 8) and the Brier score (*n* = 1). In studies reporting calibration metrics, all but two models were reported to be well-calibrated. Henriksen and colleagues (52) reported poor calibration for both included models.

### Risk of bias

The main sources of bias were identified in the “patient selection” and “index test” domains (Supplementary Fig. S1). Fifty-three studies were flagged as being at high risk of bias for the patient selection domain due to the use of a case–control design. Furthermore, within the index test domain, 72 studies were flagged as being potentially at high risk of bias due to failing to predefine the tool threshold. Overall, the risk of bias for the “reference standard” and “flow and timing” domains was judged to be low. However, 20 studies were judged to be potentially at high risk of bias in the “flow and timing” domain as not all patients were included in some analyses. The applicability of the risk of bias was judged to be low for most studies.

## Discussion

We conducted a systematic review of diagnostic risk prediction models for UGI cancers, including gallbladder, gastric, esophageal, and pancreatic cancers. This review identified 82 studies describing 162 risk prediction models, several of which had been externally validated and demonstrated good performance. To our knowledge, this is the first comprehensive systematic review of risk prediction models across UGI cancers.

Most models in this review were developed using traditional statistical methods, primarily logistic regression. However, there has been an increase in ML approaches such as neural networks and random forest since 2019—reflecting the broader adoption of ML in risk prediction research. ML offers potential advantages, including improved predictive accuracy through capturing complex, nonlinear relationships among predictors. However, these methods also pose challenges, requiring larger datasets, advanced computational resources, and specialized expertise in model tuning and validation. In this review, most externally validated models showed moderate-to-strong performance, regardless of methodological approach. Few studies compared the performance of models developed with conventional versus artificial intelligence (AI) methods (37, 49, 55, 62, 63, 66, 75, 91). Although two found their ML approach outperformed conventional approaches (55, 62), the rest reported comparable performance. Although few studies, and only two external validation studies, directly compared ML and conventional statistical models, such comparisons could provide valuable insights. Future research should focus on comparing model performance across methods and evaluating their interpretability and clinical utility.

Most models in this review were developed to detect a single UGI cancer, likely reflecting the structure of clinical guidelines, screening programs, and diagnosis pathways, which are generally cancer-site specific (99). This single-cancer approach in risk prediction model development could limit the potential for multicancer detection (MCD) approaches. To address the limitations of site-specific pathways, several countries, including Denmark and the UK, have introduced nonspecific symptom pathways, which adopt a multicancer approach for patients presenting with symptoms, which do not clearly indicate a single cancer type. The development of blood-based MCD tests, such as those used in the NHS-Galleri trial (100), further highlights this shift, especially for UGI cancers in which overlapping risk factors and nonspecific symptoms can complicate diagnosis.

Across the reviewed models, 366 unique variables were identified. Age, sex, and alcohol consumption were frequently included across models for each UGI cancer type. Ethnicity, smoking, and BMI were included in at least one model for all UGI cancers except gallbladder. These findings align with previous studies noting the prominence of age, sex, BMI, alcohol, and smoking in pancreatic and esophageal cancer models (23, 24). Laboratory tests were commonly included in models across all UGI cancers except gastroesophageal cancer. However, notable differences in variable inclusion were also evident across cancer types. For example, imaging variables were frequently included in gallbladder cancer models but were rarely included in models for other UGI cancers, whereas symptoms were consistently included in gastroesophageal models but absent from gallbladder and gastric models.

Several variables were commonly included across high-performing externally validated models. For example, hospital-based gastric cancer models with the highest AUCs included sex and prior *H*. *pylori* infection (91, 94)—a finding consistent with another review (25). Similarly, high-performing hospital-based esophageal cancer models included age, sex, and smoking (37, 61), whereas the top-performing pancreatic models in population settings incorporated comorbidities such as pancreatitis and neoplasms of the digestive system (89, 90).

The overlap of common variables across UGI cancers underscores the potential for MCD strategies. Shared variables such as demographics (age, sex, BMI, ethnicity), lifestyle (alcohol, smoking), and symptoms could be used in MCD models to identify patients at high risk of several UGI cancers. Given that diagnostic pathways for UGI cancers frequently overlap, MCD models could streamline current processes and improve resource efficiency. They could also help increase the pretest probability for patients undergoing MCD tests. However, MCD models should balance shared variables with cancer-specific predictors (such as *H*. *pylori* infection for gastric cancer) to ensure robust performance across various UGI cancers.

Consistent with previous research (23–25), we found that a minority of prediction models (20%) had been externally validated, and few studies reported calibration metrics. It is well documented that model performance often declines when models are applied to external datasets. In this review, we found that while 82.7% of the models reviewed reported AUC values greater than 0.80, less than half of externally validated models reported AUCs ≥0.80. This highlights the importance of external validation in ensuring that risk models maintain robust performance prior to clinical implementation.

Nevertheless, some externally validated models demonstrated strong performance. For example, the esophageal cancer model of Gao and colleagues (37) reported an AUC of 0.96, whereas Luan and colleagues (35) described a multicancer prediction model with an AUC of 0.91 for pancreatic cancer and 0.82 for gastric cancer. These examples demonstrate that certain models can retain high performance in external validation, highlighting the potential of some models for real-world implementation.

Models identified in this review were developed primarily to support UGI risk assessment in two contexts: asymptomatic screening and triage of symptomatic patients. Although evidence in most countries does not support screening for UGI cancers in average-risk individuals, there is evidence that screening for pancreatic cancer in higher-risk groups and for gastric cancer in higher-incidence populations may improve survival or reduce mortality (12, 101). Models could be used to identify individuals at higher risk of UGI cancers for targeted screening tests. This approach is exemplified by the externally validated ML model of Gao and colleagues (37), designed to select patients at higher risk of esophageal cancer for screening via endoscopy, and by the ENDPAC model developed by Sharma and colleagues (21), which is currently being used in clinical trials to select higher-risk individuals with new-onset diabetes for pancreatic screening with CT (22). The deep learning algorithm of Placido and colleagues (89) offers an alternative approach, which is intended to identify higher-risk individuals for screening based on complex healthcare patterns (disease trajectories) within longitudinal healthcare records. By contrast, the multicancer OncoSeek algorithm of Luan and colleagues (89), the only externally validated multicancer prediction model identified in this review, was developed to identify asymptomatic patients at elevated risk of multiple cancers (including pancreatic and esophageal), providing a potential alternative to single-site screening strategies. In patients presenting to health care with nonspecific symptoms, models identified in this study could be used to estimate the risk of one or more UGI cancers to determine whether further cancer investigation is warranted or to triage patients for urgent investigation. This is exemplified by Ho and colleagues’ (61) model designed to triage patients with symptoms of possible esophageal cancer for endoscopy and the pancreatic and gastroesophageal models of Hippisley-Cox and colleagues (59, 60) (available within consolidated QCancer clinical tools in the United Kingdom), designed to identify symptomatic patients in primary care for further investigation in line with national guidelines.

This systematic review is, to the best of our knowledge, the first to examine risk prediction models across multiple UGI cancers, rather than focusing on individual cancers. However, it is not without limitations. We employed a broad search strategy to capture a wide range of studies. Although this approach enabled a comprehensive assessment of the literature, the resulting heterogeneity in the included studies meant it was not possible to perform a meta-analysis. Additionally, although we identified overlapping variables between models, inclusion frequency does not necessarily equate to clinical importance or predictive power as model variables are often selected based on the study aims and research interests of the authors. Different subtypes of UGI cancer, such as pancreatic adenocarcinoma and pancreatic neuroendocrine tumors, may have different risk factors and may present in different ways, and their detection has different clinical implications for patients. However, a minority of validated studies provided detailed information on the pathological breakdown of included tumors or pathology-specific performance of models, which hampers the comparison of model performance and clinical implementation.

This review has several clinical implications for developing, validating, and implementing risk prediction models for UGI cancers. First, the limited external validation and inconsistent reporting of calibration highlight a critical gap in the literature. For risk prediction models to be clinically useful, they must undergo rigorous external validation in populations that reflect real-world clinical settings, including primary care, to ensure they are both accurate and generalizable to diverse patient populations (102). Furthermore, the lack of comparative studies on externally validated models for the same UGI cancers complicates the selection of clinically viable models. Future validation studies should aim to compare the performance of new models against that of established, high-performing models.

Integration into clinical workflows is another essential consideration. If models are not easily integrated, they may face adoption barriers, regardless of their accuracy (103). Previous studies and reviews have highlighted significant barriers to the implementation and clinical adoption of prediction models (105–111), including those for UGI cancers (104). The implementation of cancer tools and systems is hindered by a range of barriers at the point of care and within wider healthcare systems. These include regularity hurdles (as clinical prediction models require regulatory approval in many jurisdictions; refs. 107–109), software issues (including integrating models within clinical information technology (IT) systems; (106–108), challenges around integration into clinical workflow, and acceptability and trust issues about model predictions among clinicians (105, 110). Widely used tools, such as QCancer tools in the United Kingdom, are generally integrated into IT systems and aligned with national referral guidelines. Despite this, clinicians have identified barriers to QCancer use, including the time required to use the tools (104, 105).

Although the performance of several AI-based models identified in this review is promising, the implementation of such models presents additional challenges, and we are not aware of any AI-based UGI prediction models currently approved for use in clinical practice. Such models may be computationally intensive, and their application in many healthcare settings would require substantial investment and development in secure IT systems (111). Concerns also exist about AI explainability and how to include information from AI prediction models into shared decision-making (112). Further consideration must also be given to how models, such as the promising deep learning model of Placedo and colleagues (89), which utilizes longitudinal healthcare records, could be integrated with national screening programs, which are often run at a regional or national level. The focus of this review was not specifically on clinical implementation, but we noted that although some included studies acknowledged challenges to implementation, there was limited discussion on how these challenges could be addressed. Concerningly, some models require data that may not be routinely available in clinical settings, which could limit utility. A systematic review is currently underway to provide a framework for prediction model development and implementation research (113), including recommendations for developers, healthcare teams, and policymakers.

A key concern is the potential for risk prediction models to exacerbate existing health inequalities. Data used to develop models can often be skewed by existing structural and social inequalities, and applying these models to practice could exacerbate existing health inequalities (114). Only two studies examined performance differences between population groups (e.g., by sex and ethnicity). Most studies in this review were conducted predominantly in White or Asian populations (specifically within China). Inclusive model design and evaluation are essential in preventing the exacerbation of ethnic, socioeconomic, rural–urban, and other existing disparities in cancer detection and outcomes.

### Conclusion

In conclusion, this review identified a large number of UGI cancer risk prediction models, most of which predicted a single UGI cancer. Although some variables overlapped across different UGI cancers, others were cancer-specific—suggesting that multicancer early detection models would benefit from including both shared and unique risk factors. ML models have become more common in recent years although their performance appears generally comparable to conventional statistical models—underscoring the need for head-to-head comparative studies to evaluate performance differences. A significant limitation observed in the field is the lack of external validation. Nonetheless, several externally validated models demonstrated promising performance and merit further investigation for clinical application. Future research should ensure that UGI cancer prediction models are developed using data from the population in which they are intended to be used and that they are externally validated in diverse populations and their performance compared head-to-head (“benchmarked”) against the best-performing existing models. It is critical that developers consider user acceptability and how their models can support clinical workflow at an early stage. Given the complexity of new AI-based models, early consideration of how models can be integrated within healthcare IT systems is also imperative to ensure that translation into clinical practice is achievable.

## Supplementary Material

Supplementary Table 1 Supplementary Table 1 shows review search terms

Supplementary Table 2 Supplementary Table 2 provides details on all included studies

Supplementary Table 3 Supplementary Table 3 lists all variables included in identified models

Supplementary Table 4 Supplementary Table 4 provides details on variables within the top 15 performing models

Supplementary Figure 1 Supplementary Figure 1 shows the risk of bias assessment of included studies
