# Supplementary material for: Diagnostic Risk Prediction Models for Upper Gastrointestinal Cancers: A Systematic Review
Source: Cancer Epidemiol Biomarkers Prev. 2025 May 22;34(8):1240–51. doi: 10.1158/1055-9965.EPI-24-1714 (PMC12314510; doi:10.1158/1055-9965.EPI-24-1714)
Supplement: Supplementary Table 1 — shows review search terms [file epi-24-1714_supplementary_table_1_suppst1.docx]

Supplementary Table 1: Full search terms

| 1. | exp biliary tract neoplasms/ or gastrointestinal neoplasms/ or exp esophageal neoplasms/ or exp stomach neoplasms/ or exp pancreatic neoplasms/ or exp Esophageal Squamous Cell Carcinoma/ |
| --- | --- |
| 2. | ((gastric or stomach or $esophag* or pancrea* or intra$abdominal or gallbladder or biliary or bile duct* or gastro$intestinal or gastro$intestinal stromal) adj2 (cancer* or neoplasm* or tumo$r* or carcinoma* or lymphoma* or squamous cell carcinoma* or ductal adenocarcinoma or ductal carcinoma or adenocarcinoma* or cholangiocarcinoma or malignan*)).tw |
| 3. | 1 or 2 |
| 4. | exp "Early Detection of Cancer"/ |
| 5. | (diagnos* or detect*).tw,kw,kf. |
| 6. | 4 or 5 |
| 7. | exp Risk/ or exp Probability/ |
| 8. | exp Models, Statistical/ or exp Algorithms/ |
| 9. | 7 and 8 |
| 10. | ((risk* or probabilit* or predict*) adj4 (model* or algorithm* or tool* or index* or score* or rule* or machine learning or deep learning or calculat*)).tw. |
| 11. | 9 or 10 |
| 12. | 6 and 11 |
| 13. | 3 and 12 |
| 14. | (letter or editorial or review or conference abstract or conference review or preprint).pt. |
| 15. | 13 not 14 |
| 16. | limit 15 to yr="2000 -Current" |
