# Supplementary material for: Diagnostic Risk Prediction Models for Upper Gastrointestinal Cancers: A Systematic Review
Source: Cancer Epidemiol Biomarkers Prev. 2025 May 22;34(8):1240–51. doi: 10.1158/1055-9965.EPI-24-1714 (PMC12314510; doi:10.1158/1055-9965.EPI-24-1714)
Supplement: Supplementary Table 2 — provides details on all included studies [file epi-24-1714_supplementary_table_2_suppst2.docx]

Supplementary Table 2: Table of all included studies

| **Lead author (year)** | **Outcome** | **Model development** | **Validation method** | **Variable categories included in model** | | | | | | | | | | | | **AUC (95% CI)*** |
| --- | --- | --- | --- | --- | --- | --- | --- | --- | --- | --- | --- | --- | --- | --- | --- | --- |
|  |  |  |  | **D** | **C** | **Sx** | **Img** | **Lab** | **LS** | **Bio** | **Gen** | **GH** | **FH** | **Med** | **Oth** |  |
| Chang (2013) | OC | LR | Bootstrap | ● |  |  |  |  | ● |  |  |  |  |  |  | 0.64 (0.61, 0.66) |
|  | OC | LR | Bootstrap | ● |  |  |  |  | ● |  | ● |  |  |  |  | 0.70 (0.68, 0.72) |
| Chen (2021) | PC | XGBoost | Split-sample | ● | ● | ● | ● | ● |  |  |  | ● |  | ● |  | 0.84 (0.83, 0.85) |
| Debernardi (2023) | PC | LR | Cross-validation | ● |  |  |  | ● |  | ● |  |  |  |  |  | 0.79 (0.70, 0.89) |
|  | PC | LR | Cross-validation | ● |  |  |  | ● |  | ● |  |  |  |  |  | 0.83 (0.73, 0.89) |
|  | PC | LR | Cross-validation | ● |  |  |  | ● |  | ● |  |  |  |  |  | 0.69 (0.56, 0.81) |
|  | PC | LR | Cross-validation | ● | ● |  |  | ● |  | ● |  |  |  |  |  | 0.71 (0.61, 0.82) |
|  | PC | LR | Cross-validation | ● | ● |  |  | ● |  | ● |  |  |  |  |  | 0.71 (0.60, 0.83) |
|  | PC | LR | Cross-validation | ● | ● |  |  | ● |  | ● |  |  |  |  |  | 0.82 (0.72, 0.93) |
|  | PC | LR | Cross-validation | ● | ● |  |  | ● |  | ● |  |  |  |  |  | 0.82 (0.72, 0.93) |
| Dong (2018) | OC | LR | Bootstrap | ● |  |  |  |  | ● |  |  |  |  | ● |  | 0.79 (0.70, 0.89) |
|  | OC | LR | Bootstrap |  |  | ● |  |  |  |  |  |  |  |  |  | 0.83 (0.73, 0.89) |
|  | OC | LR | Bootstrap | ● |  | ● |  |  | ● |  |  |  |  | ● |  | 0.69 (0.56, 0.81) |
|  | OC | LR | Bootstrap | ● |  |  |  |  | ● |  | ● |  |  | ● |  | 0.71 (0.61, 0.82) |
|  | OC | LR | Bootstrap |  |  | ● |  |  |  |  | ● |  |  |  |  | 0.71 (0.60, 0.83) |
|  | OC | LR | Bootstrap | ● |  | ● |  |  | ● |  | ● |  |  | ● |  | 0.82 (0.72, 0.93) |
| Holmberg (2019) | OC | LR | None | ● |  |  | ● |  |  |  |  |  |  |  |  | 0.71 (NR) |
| Hu (2021) | GC | LR | External | ● |  |  |  | ● | ● |  |  |  |  |  |  | 0.71 (0.64, 0.77) |
| Khan (2023) | PC | XGBoost | Split-sample | ● | ● | ● |  | ● |  |  |  |  |  | ● |  | 0.80 (0.76, 0.85) |
| Bourse | PC | LR | Split-sample | ● |  |  |  | ● | ● |  |  |  |  | ● |  | 0.63 (0.57, 0.70) |
| ndpac | PC | LR | Split-sample | ● |  |  |  | ● |  |  |  | ● |  |  |  | 0.68 (0.61, 0.74) |
| Lee (2022) | PC | LR | Split-sample |  | ● | ● |  |  |  |  |  |  |  |  |  | 0.76 (NR) |
|  | PC | Voting ensemble | Split-sample |  | ● | ● |  |  |  |  |  |  |  |  |  | 0.75 (NR) |
|  | PC | Stacking | Split-sample |  | ● | ● |  |  |  |  |  |  |  |  |  | 0.74 (NR) |
|  | PC | Neural network | Split-sample |  | ● | ● |  |  |  |  |  |  |  |  |  | 0.73 (NR) |
|  | PC | LR | Split-sample |  | ● | ● |  |  |  |  |  |  |  |  |  | 0.76 (NR) |
|  | PC | Voting ensemble | Split-sample |  | ● | ● |  |  |  |  |  |  |  |  |  | 0.76 (NR) |
|  | PC | Stacking | Split-sample |  | ● | ● |  |  |  |  |  |  |  |  |  | 0.73 (NR) |
|  | PC | Neural network | Split-sample |  | ● | ● |  |  |  |  |  |  |  |  |  | 0.71 (NR) |
|  | PC | LR | Split-sample |  | ● | ● |  |  |  |  |  |  |  |  |  | 0.77 (NR) |
|  | PC | Voting ensemble | Split-sample |  | ● | ● |  |  |  |  |  |  |  |  |  | 0.73 (NR) |
|  | PC | Stacking | Split-sample |  | ● | ● |  |  |  |  |  |  |  |  |  | 0.72 (NR) |
|  | PC | Neural network | Split-sample |  | ● | ● |  |  |  |  |  |  |  |  |  | 0.71 (NR) |
| Li (2021) | OC | LR | External | ● | ● | ● |  |  | ● |  |  |  | ● |  |  | 0.80 (0.75, 0.84) |
| Liu. M (2022) | OC | LR | External | ● |  |  |  |  | ● |  |  |  | ● |  |  | 0.72 (0.72, 0.79) |
|  | OC | LR | External | ● |  |  |  |  | ● |  |  |  | ● |  |  | 0.71 (0.65, 0.78) |
| Mohammadnezhad (2023) | GC | Gradient boosting | Split-sample | ● | ● |  |  | ● | ● |  |  |  |  | ● |  | 0.98 (NR) |
| Muhammad (2019) | PC | Neural network | Cross-validation | ● | ● |  |  |  | ● |  |  |  | ● |  |  | 0.85 (0.85, 0.87) |
| Placido (2023) | PC | Bag-of-words | Split-sample (36 months) |  | ● | ● |  | ● |  |  |  |  |  |  |  | 0.81 (0.81, 0.81) |
|  | PC | MLP | Split-sample (36 months) |  | ● | ● |  | ● |  |  |  |  |  |  |  | 0.85 (0.84, 0.85) |
|  | PC | GRU | Split-sample (36 months) |  | ● | ● |  | ● |  |  |  |  |  |  |  | 0.85 (0.84, 0.85) |
|  | PC | Neural network | Split-sample  36 months) |  | ● | ● |  | ● |  |  |  |  |  |  |  | 0.88 (0.88, 0.88) |
|  | PC | Neural network | Split-sample  12 months) |  | ● | ● |  | ● |  |  |  |  |  |  |  | 0.91 (0.91, 0.91) |
|  | PC | Neural network | Split-sample  (6 months) |  | ● | ● |  | ● |  |  |  |  |  |  |  | 0.92 (0.92, 0.93) |
|  | PC | Neural network | External (36 months) |  | ● | ● |  | ● |  |  |  |  |  |  |  | 0.71 (0.71, 0.71) |
|  | PC | Neural network | External (12 months) |  | ● | ● |  | ● |  |  |  |  |  |  |  | 0.79 (0.79, 0.79) |
|  | PC | Neural network | External (6 months) |  | ● | ● |  | ● |  |  |  |  |  |  |  | 0.83 (0.83, 0.84) |
| Rubenstein (2023) | PC | LR | Split-sample | ● | ● | ● |  | ● | ● |  |  |  |  | ● |  | 0.77 (0.76, 0.78) |
| Salvatore (2021) | PC | GLM | External | ● |  |  |  |  |  |  | ● |  |  |  |  | 0.69 (0.67, 0.71) |
|  | PC | GLM | External | ● |  |  |  |  | ● |  | ● |  |  |  |  | 0.69 (0.68, 0.71) |
|  | PC | GLM | External | ● |  |  |  |  | ● |  | ● |  |  |  |  | 0.71 (0.69, 0.73) |
|  | PC | GLM | External |  | ● |  |  |  |  |  |  |  |  |  |  | 0.70 (0.69, 0.73) |
|  | PC | GLM | External | ● | ● |  |  |  | ● |  | ● |  |  |  |  | 0.81 (0.79, 0.82) |
|  | PC | GLM | External |  | ● |  |  |  |  |  | ● |  |  |  |  | 0.74 (0.72, 0.77) |
|  | PC | GLM | External | ● | ● |  |  |  | ● |  | ● |  |  |  |  | 0.81 (0.79, 0.83) |
| Sharma (2022) | PC | LR | None | ● | ● |  |  |  |  |  |  |  | ● |  |  | 0.79 (0.75, 0.83) |
|  | PC | LR | None | ● | ● |  |  |  |  |  | ● |  | ● |  |  | 0.83 (0.80, 0.86) |
| Yang (2021) | OC | LR | None | ● |  |  |  |  | ● |  |  |  | ● |  |  | 0.81 (0.79, 0.84) |
|  | OC | LR | None | ● |  |  |  |  | ● |  |  |  | ● |  |  | 0.88 (0.85, 0.90) |
| Briggs, 2022, UK | G-O | SVM | Cross-validation | ● |  | ● |  | ● |  |  |  |  |  |  |  | 0.87 (NR) |
|  | G-O | LR | Cross-validation | ● |  | ● |  | ● |  |  |  |  |  |  |  | 0.87 (NR) |
|  | G-O | RF | Cross-validation | ● |  | ● |  | ● |  |  |  |  |  |  |  | 0.86 (NR) |
| Hippisley-Cox (2011) | G-O | Cox regression | Split-sample |  |  | ● |  | ● | ● |  |  |  |  |  |  | 0.92 (0.91, 0.93) |
| Hippisley-Cox (2011) | G-O | Cox regression | Split-sample |  |  | ● |  | ● | ● |  |  |  |  |  |  | 0.89 (0.87, 0.91) |
| Hippisley-Cox (2012) | PC | Cox regression | Split-sample |  | ● | ● |  |  | ● |  |  |  |  |  |  | 0.87 (0.85, 0.88) |
| Hippisley-Cox (2012) | PC | Cox regression | Split-sample |  | ● | ● |  |  | ● |  |  |  |  |  |  | 0.84 (0.82, 0.86) |
| Hippisley-Cox, (2013a) | G-O | Cox regression | Split-sample |  |  | ● |  | ● | ● |  |  |  |  |  |  | 0.90 (0.89, 0.92) |
| Hippisley-Cox, (2013a) | PC | Cox regression | Split-sample |  |  | ● |  | ● | ● |  |  |  |  |  |  | 0.87 (0.85, 0.89) |
| Hippisley-Cox (2013b) | G-O | Cox regression | Split-sample |  |  | ● |  | ● | ● |  |  |  |  |  |  | 0.93 (0.92, 0.93) |
| Hippisley-Cox (2013b) | PC | Cox regression | Split-sample |  |  | ● |  | ● | ● |  |  |  |  |  |  | 0.89 (0.87, 0.91) |
| Ahmadi (2020) | OC | LR | Bootstrap | ● | ● | ● |  |  |  |  |  |  |  |  |  | 0.82 (0.77, 0.87) |
| Ahmed (2018) | PC | LR | Bootstrap | ● | ● | ● | ● |  |  |  |  |  |  |  |  | 0.95 (NR) |
| Blyuss (2020) | PC | LR | Split-sample | ● |  |  |  | ● |  | ● |  |  |  |  |  | 0.94 (0.91, 0.97) |
|  | PC | Neural network | Split-sample | ● |  |  |  | ● |  | ● |  |  |  |  |  | 0.93 (0.90, 0.97) |
|  | PC | Neuro-fuzzy | Split-sample | ● |  |  |  | ● |  | ● |  |  |  |  |  | 0.94 (0.91, 0.97) |
| Cai (2011) | PC | LR | None | ● |  |  | ● | ● |  |  |  |  |  |  |  | 0.81 (0.70, 0.91) |
|  | PC | LR | Bootstrap | ● |  |  | ● | ● |  |  |  |  |  |  |  | 0.72 (0.66, 0.78) |
| Cai (2019) | GC | LR | Split-sample | ● |  |  |  | ● | ● |  |  |  |  |  |  | 0.73 (0.68, 0.77) |
| Chakraborty (2021) | GC | LR | None | ● |  |  |  |  | ● |  |  |  |  |  |  | 0.91 (0.86, 0.94) |
| Corona (2018) | GC | LR | External | ● |  |  |  | ● |  | ● |  |  |  |  |  | 0.86 (0.78, 0.91) |
| Crouwel (2018) | G-O | LR | Bootstrap | ● |  | ● |  | ● |  |  |  |  |  |  |  | 0.87 (NR) |
| Etemadi (2012) | OC | LR | Cross-validation | ● | ● |  |  |  | ● |  |  | ● | ● |  |  | 0.77 (0.74, 0.80) |
|  | OC | LR | Cross-validation | ● | ● | ● |  |  | ● |  |  | ● | ● |  |  | 0.87 (0.85, 0.89) |
| Gao (2023) | OC | LR | None | ● | ● |  |  |  | ● |  |  | ● | ● |  | ● | 0.95 (0.94, 0.97) |
|  | OC | Adaptive boosting | Cross-validation | ● | ● |  |  |  | ● |  |  | ● | ● |  | ● | 0.95 (0.93, 0.97) |
|  | OC | LightGBM | External | ● | ● |  |  |  | ● |  |  | ● | ● |  | ● | 0.96 (0.92, 0.99) |
|  | OC | XGBoost | Cross-validation | ● | ● |  |  |  | ● |  |  | ● | ● |  | ● | 0.96 (0.94, 0.97) |
|  | OC | SVM | Cross-validation | ● | ● |  |  |  | ● |  |  | ● | ● |  | ● | 0.92 (0.89, 0.95) |
|  | OC | RF | Cross-validation | ● | ● |  |  |  | ● |  |  | ● | ● |  | ● | 0.93 (0.90, 0.95) |
| Henriksen (2016) | PC | LR | Cross-validation | ● |  |  |  |  |  |  | ● |  |  |  |  | 0.86 (0.81, 0.91) |
| Henriksen (2021) | PC | LR | External | ● |  |  |  |  |  |  | ● |  |  |  |  | 0.77 (0.69, 0.84) |
|  | PC | LR | External | ● |  |  |  | ● |  |  | ● |  |  |  |  | 0.85 (0.79, 0.91) |
| Ho (2023) | OC | LR | Cross-validation | ● | ● | ● |  |  | ● |  |  |  |  |  |  | 0.71 (0.61, 0.81) |
|  | OC | LR | External | ● | ● | ● |  |  | ● |  |  |  |  |  |  | 0.92 (0.88, 0.96) |
| Huang (2022) | GC | SVM | Cross-validation | ● | ● |  |  | ● |  |  |  |  |  |  |  | 0.85 (0.80, 0.91) |
|  | GC | RF | External | ● | ● |  |  | ● |  |  |  |  |  |  |  | 0.74 (NR) |
|  | GC | K-nearest neighbours | External | ● | ● |  |  | ● |  |  |  |  |  |  |  | 0.75 (NR) |
|  | GC | Penalised lasso | External | ● | ● |  |  | ● | ● |  |  |  |  |  |  | 0.82 (NR) |
|  | GC | LR | External | ● | ● |  |  | ● | ● |  |  |  |  |  |  | 0.81 (NR) |
| Hsu (2012) | G-O | LR | None | ● |  | ● |  |  | ● |  |  |  |  |  |  | 0.85 (0.80, 0.91) |
| In (2020) | GC | LR | None | ● | ● |  |  | ● | ● |  |  |  | ● |  |  | 0.87 (0.81, 0.93) |
|  | GC | LR | None | ● | ● |  |  | ● | ● |  |  |  | ● |  |  | 0.97 (0.95, 0.99) |
| Kamran (2022) | G-O | LR | Bootstrap | ● |  | ● |  |  |  |  |  |  |  |  |  | 0.81 (0.76, 0.85) |
|  | G-O | LR | Bootstrap | ● |  | ● |  |  |  |  |  |  |  |  |  | 0.83 (0.79, 0.87) |
| Kapoor (2005) | G-O | LR | External |  |  | ● |  | ● |  |  |  |  |  |  |  | NR (NR) |
| Khademi (2012) | G-O | LR | Cross-validation | ● |  | ● |  |  | ● |  |  |  | ● |  |  | 0.82 (0.76, 0.88) |
|  | G-O | LR | None | ● |  | ● |  |  |  |  |  |  |  |  |  | 0.82 (0.77, 0.87) |
| Kong (2020) | GC | LR | None |  |  |  |  |  | ● |  | ● |  |  |  |  | 0.75 (NR) |
| Kurita (2019) | PC | NN | Cross-validation | ● |  |  | ● |  | ● |  |  |  |  |  | ● | 0.97 (NR) |
|  | PC | NN | Cross-validation | ● |  |  | ● | ● |  |  |  |  |  |  | ● | 0.96 (NR) |
| Lee (2009) | GC | LR | Bootstrap | ● | ● |  |  |  | ● |  |  | ● | ● |  | ● | 0.90 (0.88, 0.93) |
| Lee (2012) | PC | LR | None | ● |  | ● |  |  |  |  |  |  | ● |  |  | NR (NR) |
| Lee (2023) | PC | LR | Cross-validation | ● |  |  |  | ● |  | ● |  |  |  |  |  | NR (NR) |
| Lin (2022) | GC | LR | Bootstrap | ● |  |  | ● | ● |  |  |  |  |  |  |  | 0.86 (0.79, 0.94) |
| Liu (2012) | GBC | LR | None | ● |  |  | ● |  |  |  |  |  |  |  |  | 0.92 (0.86, 0.97) |
| Liu (2017) | GC | LR | Split-sample | ● |  |  |  |  |  | ● |  |  |  |  |  | 0.81 (0.70, 0.91) |
| Liu (2022) | PC | LR | Cross-validation |  |  |  | ● | ● |  |  |  |  |  |  |  | 0.96 (0.82, 0.99) |
|  | PC | LR | Cross-validation |  |  |  |  | ● |  |  |  |  |  |  |  | 0.80 (0.61, 0.92) |
| Lu (2006) | PC | LR | None | ● | ● | ● |  |  | ● |  |  |  | ● |  |  | 0.98 (NR) |
| Luan (2023) | OC | RF | External | ● |  |  |  | ● |  |  |  |  |  |  |  | 0.81 (NR) |
|  | PC | RF | External | ● |  |  |  | ● |  |  |  |  |  |  |  | 0.91 (NR) |
|  | GC | RF | External | ● |  |  |  | ● |  |  |  |  |  |  |  | 0.82 (NR) |
| Manuel-Vazquez (2022) | PC | LR | None | ● | ● |  | ● | ● |  |  |  |  |  |  |  | NR (NR) |
| Mather (2023) | OC | LR | None |  |  | ● |  |  |  |  |  |  |  |  |  | 0.44 (NR) |
|  | OC | LR | None |  |  | ● |  |  |  |  |  |  |  |  |  | 0.44 (NR) |
|  | OC | LR | None |  |  | ● |  |  |  |  |  |  |  |  |  | 0.52 (NR) |
|  | OC | LR | None |  |  | ● |  |  |  |  |  |  |  |  |  | 0.85 (NR) |
|  | OC | LR | Cross-validation | ● |  | ● |  |  |  |  |  |  |  |  |  | 0.89 (NR) |
| Matsunaga (2017) | PC | LR | None | ● |  |  |  |  |  | ● |  |  |  |  |  | 0.82 (0.79, 0.93) |
| Nakatochi (2018) | PC | LR | Cross-validation |  |  |  |  |  | ● |  | ● |  | ● |  |  | 0.63 (0.60, 0.66) |
| Park (2022) | PC | LR | Split-sample |  |  |  |  | ● |  |  |  |  |  |  |  | 0.63 (0.63, 0.63) |
|  | PC | XGBoost | None |  |  |  |  | ● |  |  |  |  |  |  |  | 0.66 (0.66, 0.66) |
|  | PC | NN | Split-sample |  |  |  |  | ● |  |  |  |  |  |  |  | 0.83 (0.82, 0.84) |
|  | PC | NN | Split-sample |  |  |  |  | ● |  |  |  |  |  |  |  | 0.83 (0.82, 0.84) |
|  | PC | NN | Split-sample |  |  |  |  | ● |  |  |  |  |  |  |  | 0.82 (0.80, 0.84) |
| Qiu (2020) | GC | LR | Cross-validation | ● |  |  |  |  |  |  | ● |  |  |  |  | 0.68 (NR) |
| Qu (2023) | PC | LR | Split-sample | ● |  |  | ● | ● |  |  |  |  |  |  |  | 0.76 (NR) |
|  | PC | LR | Split-sample | ● |  |  | ● | ● |  |  |  |  |  |  |  | 0.83 (NR) |
|  | PC | LR | Split-sample | ● |  |  | ● | ● |  |  |  |  |  |  |  | 0.88 (NR) |
| Saukkonen (2018) | PC | LR | None | ● |  |  |  | ● |  | ● |  |  |  |  |  | 0.87 (0.80, 0.93) |
| Sharma (2018) | PC | LR | None |  |  |  |  | ● |  |  |  | ● |  |  |  | 0.87 (NR) |
| Shen (2013) | PC | LR | External |  |  |  | ● | ● |  |  |  |  |  |  |  | 0.94 (NR) |
| Shen (2020) | OC | LR | Cross-validation | ● |  |  |  |  | ● |  |  |  |  |  |  | 0.77 (0.74, 0.81) |
| Tao. J (2020) | GBC | LR | None | ● |  |  | ● |  |  |  |  |  |  |  |  | 0.88 (NR) |
| Tao. W (2020) | GC | LR | External | ● | ● |  |  | ● | ● |  |  |  |  |  |  | 0.88 (0.85, 0.92) |
| Tox (2007) | PC | LR | None | ● |  |  | ● |  |  |  |  |  |  |  |  | 0.85 (NR) |
| Wang (2022) | PC | LR | None | ● |  |  |  | ● |  |  |  |  |  |  |  | 0.96 (0.92, 0.99) |
| Xiang (2023) | GBC | LR | Split-sample | ● |  |  | ● | ● |  |  |  |  |  |  |  | 0.84 (0.76, 0.92) |
| Yang (2014) | PC | LR | Split-sample | ● | ● |  |  | ● |  |  |  |  |  |  |  | 0.81 (0.76, 0.86) |
| Yokoyama (2008) | OC | LR | Cross-validation |  |  |  |  |  | ● |  | ● |  |  |  |  | 0.86 (NR) |
|  | OC | LR | Cross-validation |  |  |  |  |  | ● |  |  |  |  |  |  | 0.84 (NR) |
| Yokoyama (2013) | OC | LR | External |  |  |  |  |  | ● |  |  |  |  |  |  | NR |
| Zardab (2023) | PC | LR | Cross-validation | ● | ● | ● |  | ● |  |  |  |  |  |  |  | 0.89 (0.84, 0.94) |
|  | PC | LR | Cross-validation | ● | ● | ● |  | ● |  |  |  |  |  |  |  | 0.90 (0.85, 0.94) |
|  | PC | Ridge regression | Cross-validation | ● | ● | ● |  | ● |  |  |  |  |  |  |  | 0.89 (0.85, 0.94) |
|  | PC | LASSO | Cross-validation | ● | ● | ● |  | ● |  |  |  |  |  |  |  | 0.89 (0.84, 0.94) |
|  | PC | Elastic net | Cross-validation | ● | ● | ● |  | ● |  |  |  |  |  |  |  | 0.89 (0.85, 0.94) |
| Zhang (2015) | GBC | LR | None | ● |  |  |  | ● |  |  |  |  |  |  |  | 0.79 (NR) |
| Zhang (2018) | PC | LR | Cross-validation | ● |  |  | ● | ● |  |  |  |  |  |  |  | 0.69 (NR) |
| Zhang (2022) | PC | LR | Split-sample | ● | ● | ● | ● | ● |  |  |  |  |  |  |  | 0.80 (0.65, 0.96) |
| Zhang (2023) | GC | LR | External | ● |  |  |  | ● | ● |  |  |  | ● |  |  | 0.60 (NR) |
| Zhou (2016) | GBC | LR | External | ● | ● |  | ● |  | ● |  |  |  |  |  |  | 0.86 (NR) |
| Zhou (2021) | GC | LR | External | ● |  |  |  |  | ● |  |  |  | ● |  |  | 0.76 (0.71, 0.82) |
| Zhou (2022) | GC | LR | External | ● |  |  |  | ● |  |  |  |  |  |  |  | 0.79 (NR) |
| Zhu, S (2020) | GC | Gradient boosting | Split-sample | ● |  |  |  | ● |  |  |  |  |  |  |  | 0.91 (NR) |
| Zhu, Z (2023) | GBC | LR | External | ● | ● |  | ● | ● |  |  |  |  |  |  |  | 0.88 (0.82, 0.94) |
| Zhu. Q, 2023, China | GC | LR | None | ● |  |  |  |  |  | ● |  |  |  |  |  | 0.86 (0.80, 0.90) |
|  | GC | LR | None | ● |  |  |  |  |  | ● |  |  |  |  |  | 0.87 (0.82, 0.91) |

Abbreviations: AUC, area under the curve; C, comorbidities; CI; confidence interval; D, demographics; GBC, gallbladder cancer; GC, gastric cancer; Gen, genetics; GH, general health; GLM, generalised linear model; G-O, gastro-oesophageal cancer; FH, family history; Img, imaging; Lab, lab test; LR, logistic regression; LS, lifestyle; NR, not reported; Oth, other; PC, pancreatic cancer; Sx, symptom
