# Supplementary material for: Diagnostic Risk Prediction Models for Upper Gastrointestinal Cancers: A Systematic Review
Source: Cancer Epidemiol Biomarkers Prev. 2025 May 22;34(8):1240–51. doi: 10.1158/1055-9965.EPI-24-1714 (PMC12314510; doi:10.1158/1055-9965.EPI-24-1714)
Supplement: Supplementary Table 3 — lists all variables included in identified models [file epi-24-1714_supplementary_table_3_suppst3.docx]

Supplementary Table 3: List of all unique variables included in all models by variable category

| **Variable Category** | **Variable** |
| --- | --- |
| biomarker | C16 metabolites |
| biomarker | gastric juice pH |
| biomarker | LRG1 |
| biomarker | LYVE1 |
| biomarker | NPM1 |
| biomarker | PGK1 |
| biomarker | phenylalanine |
| biomarker | RAE1 |
| biomarker | REG1B |
| biomarker | REG4 |
| biomarker | S100P |
| biomarker | SM(OH)22:1 |
| biomarker | TFF1 |
| biomarker | tryptophan |
| biomarker | TTR |
| biomarker | tyrosine |
| comorbidities | acute and subacute necrosis of the liver |
| comorbidities | acute bronchitis and bronchiolitis |
| comorbidities | acute hepatitis |
| comorbidities | acute laryngitis |
| comorbidities | acute nasopharyngitis |
| comorbidities | anaemia |
| comorbidities | anorexia |
| comorbidities | atrophic gallbladder |
| comorbidities | bacterial pneumonia |
| comorbidities | benign neoplasm of colon, rectum, anus |
| comorbidities | biliary tract disease |
| comorbidities | calculus of kidney and ureter |
| comorbidities | cancer history |
| comorbidities | cancer of other lymphoid/histiocytic tissue |
| comorbidities | cardiac dysrhythmias |
| comorbidities | cerebral artery occlusion with cerebral infarction |
| comorbidities | cerebrovascular disease |
| comorbidities | CHD |
| comorbidities | cholangitis |
| comorbidities | cholecystitis |
| comorbidities | cholelithiasis |
| comorbidities | chronic liver disease and cirrhosis |
| comorbidities | CKD |
| comorbidities | colon cancer |
| comorbidities | congestive heart failure |
| comorbidities | connective tissue disease |
| comorbidities | COPD |
| comorbidities | coronary artery disease |
| comorbidities | course of gallstones |
| comorbidities | delirium |
| comorbidities | dementia |
| comorbidities | diabetes |
| comorbidities | diabetes mellitus |
| comorbidities | diabetes type 2 |
| comorbidities | diabetes with complications |
| comorbidities | diseases of pancreas |
| comorbidities | dislocation, sprain of joints and ligaments in head |
| comorbidities | disorders of function of stomach |
| comorbidities | disorders of lipid metabolism |
| comorbidities | duodenal ulcer |
| comorbidities | emphysema |
| comorbidities | enlarged lymph nodes |
| comorbidities | functional digestive disorders |
| comorbidities | gastric ulcer history |
| comorbidities | gastritis and duodenitis |
| comorbidities | genital prolapse |
| comorbidities | gingival and periodontal diseases |
| comorbidities | gout |
| comorbidities | heart attack |
| comorbidities | heart disease |
| comorbidities | heart failure |
| comorbidities | history of cancer |
| comorbidities | history of OC |
| comorbidities | HIV |
| comorbidities | hypertension |
| comorbidities | hypertensive heart disease |
| comorbidities | intestinal metaplasia |
| comorbidities | liver disease |
| comorbidities | malignant neoplasm of biliary tract |
| comorbidities | malignant neoplasm of bronchus and lung |
| comorbidities | malignant neoplasm of cervix |
| comorbidities | malignant neoplasm of digestive system |
| comorbidities | malignant neoplasm of gallbladder and bile ducts |
| comorbidities | malignant neoplasm of respiratory and digestive organs |
| comorbidities | malignant neoplasm of small intestine |
| comorbidities | malignant neoplasm of stomach |
| comorbidities | Medical history |
| comorbidities | mental and behavioural disorders due to use of alcohol |
| comorbidities | mental and behavioural disorders due to use of tobacco |
| comorbidities | mild liver disease |
| comorbidities | moderate/severe liver disease |
| comorbidities | myocardial infarction |
| comorbidities | neurotic disorders |
| comorbidities | oesteoporosis |
| comorbidities | opiate use |
| comorbidities | osteoarthrosis and allied disorders |
| comorbidities | other cancer |
| comorbidities | other cellulitis and abscess |
| comorbidities | other diseases of biliary tract |
| comorbidities | other diseases of gallbladder and bile duct |
| comorbidities | other diseases of oesophagus |
| comorbidities | other diseases of pancreas |
| comorbidities | other disorders of pancreatic internal secretion |
| comorbidities | other forms of chronic ischemic heart disease |
| comorbidities | other liver diseases |
| comorbidities | other nervous system disorders |
| comorbidities | other symptoms of digestive system |
| comorbidities | pancreatic disorders (not diabetes) |
| comorbidities | pancreatitis |
| comorbidities | paraplesia/hemipleasia |
| comorbidities | peptic ulcer |
| comorbidities | peptic ulcer disease |
| comorbidities | Peptic ulcer history or oesophagitis |
| comorbidities | peripheral vascular disease |
| comorbidities | phlebitis and thrombophlebitis |
| comorbidities | pneumonia unspecified |
| comorbidities | Prior h.pylori infection |
| comorbidities | psychological disorders |
| comorbidities | renal disease |
| comorbidities | renal failure |
| comorbidities | rheumatic heart disease |
| comorbidities | stroke |
| comorbidities | umbilical hernia |
| comorbidities | unspecified disorders of the back |
| comorbidities | urticaria |
| demographics | age |
| demographics | age at diagnosis of diabetes |
| demographics | age^2 |
| demographics | area per person |
| demographics | BMI |
| demographics | bmi |
| demographics | BMI 10 years prior |
| demographics | education |
| demographics | ethnicity |
| demographics | height |
| demographics | immigration |
| demographics | income |
| demographics | marital status |
| demographics | race |
| demographics | residence |
| demographics | SES |
| demographics | sex |
| demographics | smoking |
| demographics | urbanicity |
| demographics | waist circumference |
| demographics | waist-hip ratio |
| demographics | weight |
| family_history | Family history of diabetes |
| family_history | Family history of digestive cancer |
| family_history | family history of gastric ulcer |
| family_history | family history of GC |
| family_history | Family history of GC |
| family_history | family history of OC |
| family_history | Family history of OC |
| family_history | Family history of OC |
| family_history | Family history of PC |
| family_history | family history of UGI |
| family_history | Family history of UGI cancer |
| family_history | Family members over 50 with PC |
| genetic | 25 SNPs |
| genetic | ALDH2 genotype |
| genetic | genotyping array |
| genetic | hypermethylation markers |
| genetic | PRS |
| genetic | rs7158663 |
| genetic | SNPs |
| health | oral health |
| health | outpatient visits |
| health | perceived health status |
| health | tooth loss |
| health | weight change |
| health | weight loss change |
| imaging | abnormal imaging findings |
| imaging | asymmetrically thickened wall |
| imaging | connection of pancreatic duct and cyst |
| imaging | cyst type |
| imaging | dilation of main pancreatic duct |
| imaging | double-duct sign |
| imaging | gallbladder wall calcification |
| imaging | gallstone size |
| imaging | increased parenchyma echogenicity |
| imaging | intralesional vessels |
| imaging | intraluminal polypoid lesion |
| imaging | Kyoto classification |
| imaging | lesion size |
| imaging | location of cystic lesion |
| imaging | mass location |
| imaging | mass number |
| imaging | mass size |
| imaging | maximum BE length |
| imaging | mpd diameter |
| imaging | mucosal line disruption |
| imaging | mural nodules |
| imaging | nontruncated panreatic duct stenosis |
| imaging | other and unspecified benign neoplasm |
| imaging | rad_score |
| imaging | septum thickening |
| imaging | splenic vein invasion |
| imaging | suspicious lymph nodes |
| imaging | tumour diameter |
| imaging | tumour edge with pseudopodia-like extensions |
| imaging | tumour form |
| imaging | vascular infiltration |
| imaging | venous phase portal venous CT value |
| imaging | wall intactness |
| imaging | wall thickness ratio |
| imaging | washout time |
| lab test | AAT |
| lab test | AFP |
| lab test | alanine transferase |
| lab test | albumin |
| lab test | alkaline phosphates |
| lab test | AlkPhos |
| lab test | ALP |
| lab test | amylase |
| lab test | anti-hpylori |
| lab test | aspartate transferase |
| lab test | basophil automated |
| lab test | basophil count |
| lab test | bilirubin |
| lab test | blood glucose |
| lab test | blood pressure |
| lab test | blood type |
| lab test | blood urea nitrogen |
| lab test | CA125 |
| lab test | CA15-3 |
| lab test | CA199 |
| lab test | CA19-9 |
| lab test | CA72-4 |
| lab test | calcium level |
| lab test | CEA |
| lab test | change in blood glucose |
| lab test | chloride level |
| lab test | chlorine |
| lab test | cholesterol |
| lab test | CO2 |
| lab test | c-peptide |
| lab test | creatine level |
| lab test | creatinine level |
| lab test | CRP |
| lab test | CYFRA |
| lab test | direct bilirubin |
| lab test | eosinophil automated |
| lab test | eosinophil count |
| lab test | fasting blood glucose |
| lab test | G-17 |
| lab test | glucose |
| lab test | glucose white blood cell |
| lab test | H. Pylori infection |
| lab test | haematocrit |
| lab test | haemoglobin |
| lab test | HBA1C |
| lab test | HDL |
| lab test | IM |
| lab test | LDL |
| lab test | lipase measurement |
| lab test | liver function test |
| lab test | lymphocyte automated |
| lab test | lymphocyte count |
| lab test | magnesium level |
| lab test | mean corpuscular haemoglobin |
| lab test | mean corpuscular haemoglobin concentration |
| lab test | mean corpuscular haemoglobin level |
| lab test | mean corpuscular volume |
| lab test | mean platelet volume |
| lab test | monocyte automated |
| lab test | monocyte count |
| lab test | neutrophil automated |
| lab test | neutrophil count |
| lab test | neutrophil-lymphocyte ratio |
| lab test | nucleated red blood cell auto |
| lab test | PG category |
| lab test | PG I/II ratio |
| lab test | PG level |
| lab test | PG-II level |
| lab test | PGR |
| lab test | phosphorus level |
| lab test | platelet count |
| lab test | platelet volume |
| lab test | potassium level |
| lab test | pre-diabetes estimated average glucose |
| lab test | protein level |
| lab test | prothrombin time |
| lab test | red blood cell count |
| lab test | red cell diameter width |
| lab test | sodium level |
| lab test | total cholesterol |
| lab test | triglycerides |
| lab test | white cell count |
| lifestyle | acculturation |
| lifestyle | age started smoking |
| lifestyle | alcohol drinking |
| lifestyle | alcohol flushing |
| lifestyle | alcohol frequency |
| lifestyle | carcinogenic job exposure |
| lifestyle | cultural foods |
| lifestyle | eating speed |
| lifestyle | financial status |
| lifestyle | fried food |
| lifestyle | fruit and vegetable frequency |
| lifestyle | hot food preference |
| lifestyle | ingestion of leftover food |
| lifestyle | intake of hot food |
| lifestyle | meat consumption |
| lifestyle | missing and filled teeth |
| lifestyle | physical activity |
| lifestyle | pickled food |
| lifestyle | pickled/salted food |
| lifestyle | preserved foods |
| lifestyle | rapid eating |
| lifestyle | reads local newspaper |
| lifestyle | regular eating |
| lifestyle | salt consumption |
| lifestyle | salted roe consumption |
| lifestyle | smoked food consumption |
| lifestyle | smoking |
| lifestyle | smoking |
| lifestyle | smoking frequency |
| lifestyle | tea drinking |
| lifestyle | tea temperature |
| lifestyle | tooth brushing time |
| lifestyle | type of drinking water |
| lifestyle | use of coal/wood when cooking |
| lifestyle | years since quitting smoking |
| medication | ciprofloxacin |
| medication | histamine type 2 receptor antagonist |
| medication | insulin |
| medication | metformin HCL |
| medication | NSAID |
| medication | opiate use |
| medication | oral hypoglucemics |
| medication | proton pump inhibitor |
| Other | Cytological variables |
| Other | cytological variables |
| Other | gastroscopy |
| symptom | abdominal distension |
| symptom | abdominal pain |
| symptom | anaemia |
| symptom | anorexia |
| symptom | appetite loss |
| symptom | blood clots |
| symptom | butterflies feeling |
| symptom | chest pain |
| symptom | chest pain length time |
| symptom | constipation |
| symptom | digestive symptoms |
| symptom | duration of symptoms |
| symptom | dyspepsia |
| symptom | dysphagia |
| symptom | dysphagia score |
| symptom | epigastric pain |
| symptom | fatigue |
| symptom | gastrointestinal bleeding |
| symptom | general symptoms |
| symptom | GERD |
| symptom | GI bleeding |
| symptom | GI bleeding * dysphagia |
| symptom | haematemesis |
| symptom | head and neck symptoms |
| symptom | heartburn |
| symptom | heartburn/indigestion |
| symptom | indigestion |
| symptom | jaundice |
| symptom | melena |
| symptom | nausea/vomiting |
| symptom | neck lump |
| symptom | obstructive jaundice |
| symptom | oesophageal symptoms |
| symptom | pain |
| symptom | pain in throat/chest/back |
| symptom | pharyngeal symptoms |
| symptom | progressive dysphagia |
| symptom | reflux |
| symptom | regurgitation length time |
| symptom | respiratory symptoms |
| symptom | restrosternal pain/back pain/neck pain |
| symptom | sour taste frequency |
| symptom | sour taste present |
| symptom | swallowing difficulty |
| symptom | swallowing pain |
| symptom | Symptoms preventing eating/drinking |
| symptom | UGI bleeding |
| symptom | upper airway symptoms |
| symptom | vomiting |
| symptom | VTE |
| symptom | weight change |
| symptom | weight loss |
| symptom | weight loss * dysphagia |
