# Supplementary material for: Diagnostic Risk Prediction Models for Upper Gastrointestinal Cancers: A Systematic Review
Source: Cancer Epidemiol Biomarkers Prev. 2025 May 22;34(8):1240–51. doi: 10.1158/1055-9965.EPI-24-1714 (PMC12314510; doi:10.1158/1055-9965.EPI-24-1714)
Supplement: Supplementary Table 4 — provides details on variables within the top 15 performing models [file epi-24-1714_supplementary_table_4_suppst4.docx]

Supplementary Table 4: Breakdown of variables for top 15 best performing externally validated models.

| **Model name** | **Outcome** | **AUC** | **Variable selection** | **Final variables selected for model** |
| --- | --- | --- | --- | --- |
| Henriksen_2021_v2 | PDAC | 0.85 | Logistic regression with backward feature elimination approach 0.05 as the significance level for removal from the model. | **Demographic:** Age(cont.), Sex (m/f)  **Lab:** Ca 19-9 (cont.)  **Genetics:** BMP3, RASSF1A, BNC1, MESTv2, TFPI2, APC, SFRP1, SFRP2 (cfDNA hypermethylation status) |
| Luan_2023_PC | PC | 0.91 | Previous research informed protein tumour marker choice for including into AI algorithm | **Demographic:** Age (cont.), Sex (m/f)  **Lab:** AFP (>5.8 IU/ml)), CA125 (>35 U/ml), CA15-3 (>26.4 U/ml), CA19-9 (>27 U/ml), CA72-4 (>6.9 U/ml), CEA (>4.7ng/ml), CYFRA (>3.3ng/ml) |
| Placido_2023_ Transformer | PC | 0.83 | All ICD-10 codes from patient electronic health record | All ICD-10 codes from patient electronic health record |
| Salvatore_2021_v5 | PC | 0.81 | ICD 10 codes used to construct phenotype risk score | **Demographic:** Age (cont.), Sex (m/f), BMI (cont)  **Comorbidities:** Phenotype risk score constructed using time associated diagnosis codes from electronic records  **Lifestyle:** Smoking status (ever/neve), Alcohol use (ever/never) |
| Salvatore_2021_v7 | PC | 0.81 | ICD 10 codes used to construct phenotype risk score | **Demographic:** Age (cont.), Sex (m/f), BMI (cont)  **Comorbidities:** Phenotype risk score constructed using time associated diagnosis codes from electronic records  **Lifestyle:** Smoking status (ever/neve), Alcohol use (ever/never)  **Genetics:** Polygenic Risk Score |
| Gao_2023_  Light_GBM | OSCC (78%), OAC (22%) | 0.96 | Logistic regression with backward feature elimination approach | **Demographic:** Age (cont.), Sex (m/f), BMI (cont), Education level (primary/high school/college), Residence (urban/rural)  **Comorbidities:** Personal history of cancer (y/n)  **Lifestyle:** Smoking status (y/n), Alcohol use (y/n), Hot food preference (y/n), Pickled food preference (y/n)  **General:** Tooth loss (none, 1-4, 5-12. >12), Alcohol flushing (y/n)  **Family History:** Oesophageal Cancer (y/n) |
| Ho_2023 | OSCC (7%), OAC (93%) | 0.92 | Top 20 variables chosen after ranking by information gain and correlation-based selection calculated | **Demographic:** Age(cont.), Sex (m/f)  **Comorbidities:** Known Psychological disorder (y/n)  **Symptom:** Dysphagia score (0-3), Unexplained weight loss (y/n), Chest pain (none/less than 6 months/6 months to 1 year/1 year to 5 year), Swallowing pain (y/n), Sore taste present (y/n), Butterflies feeling (none/occasionally/quite often/very often/unknown)  **Lifestyle:** Ever smoker (y/n), Smoking pack years (cont.), Read local newspaper (never/rarely/sometimes/often/unknown) |
| Li 2021 | OSCC | 0.80 | Logistic regression with backward feature elimination approach 0.05 as the significance level for removal from the model. | **Demographic:** Age (categorical), Sex (m/f)  **Comorbidities:** History of peptic ulcer or oesophagitis (y/n)  **Symptoms:** Retrosternal pain, back pain, or neck pain (y/n)  **Lifestyle:** Smoking pack years (0/<30/=/>30), Consumption of salted food (low/high) and fresh fruit (low/high)  **Family history:** Upper GI cancer (y/n) |
| Luan_2023_OC | OC | 0.81 | Previous research informed protein tumour marker choice for inclusion in AI algorithm | **Demographic:** Age (cont.), Sex (m/f), AFP (>5.8 IU/ml)), CA125 (>35 U/ml), CA15-3 (>26.4 U/ml), CA19-9 (>27 U/ml), CA72-4 (>6.9 U/ml), CEA (>4.7ng/ml), CYFRA (>3.3ng/ml) |
| Huang_2022_PL | GC (non-cardia) | 0.82 | Selected by Machine Learning LASSO approach | **Demographic:** Sex (m/f), BMI (cont), Race (White/Black/Hispanic/American Indian or Alaskan native/Asian or pacific islander/other), Immigrant (y/n)  **Comorbidities:** Prior H pylori infection (y/n), Anaemia (y/n) |
| Huang_2022_LR | GC (non-cardia) | 0.81 | All considered variables included apart from age and sex as they were basis for case-control matching | **Demographic:** Race (White/Black/Hispanic/American Indian or Alaskan native/Asian or pacific islander/other), Immigrant (y/n) **Comorbidities:** Prior H pylori infection (y/n), Anaemia (y/n) **Lifestyle:** Smoking status (current/former) |
| Luan_2023_GC | GC | 0.82 | Previous research informed protein tumour marker choice for including into AI algorithm | **Demographic:** Age (cont.), Sex (m/f) **Lab:** AFP (>5.8 IU/ml)), CA125 (>35 U/ml), CA15-3 (>26.4 U/ml), CA19-9 (>27 U/ml), CA72-4 (>6.9 U/ml), CEA (>4.7ng/ml), CYFRA (>3.3ng/ml) |
| Tao_W_2020 | GC | 0.88 | Univariate analysis (cut off p<0.05) and multiple logistic regression analysis | **Demographic** Age (<=45; >45 to <=55; >55 to <=65; >65), Sex (m/f)  **Comorbidities:** H Pylori infection (yes/no) **Lab:** Pepsinogen (PG) level (PGI <=43.6 ug/L and PGR > 2.1; PGI > 43.6 and PGR > 2.1; PGI <=43.6 and PGR > 2.1)  **Lifestyle:** Type of drinking water (tap/well or cellar)  **Family history:** Gastric cancer (y/n) |
| Zhou_2016 | GBC | 0.86 | Logistic regression with backward feature elimination approach 0.05 as the significance level for removal from the model. | **Demographic**: Age (cont)  **Comorbidities:** Course of diagnosed gallstones (years)  **Imaging:** features of atrophic gallbladder (y/n), Gallbladder wall calcification (y/n), intraluminal polypoid lesion(y/n), wall thickness ratio (cont.), mucosal line disruption (y/n), **Lifestyle**: Hazardous alcohol use (y/n) |
| Zhu_Z_2023 | GBC | 0.88 | Logistic regression multivariate analysis were used to construct the weighted score for indicators. | **Demographic**: Age (<=58.5 years, > 58.5 years)  **Comorbidities:** Course of gallstones (<=10 years, > 10 years)  **Imaging:** size of gallstones (<= 1.95, > 1.95cm)  **Lab:** CEA(<=5 or />5ng/ml), CA199 (<=37 or > 37 U/mL) |

Abbreviations: cont, continuous; GBC, gallbladder cancer; GC, gastric cancer; Lab, lab tests; OSCC, oesophageal squamous cell OC oesophageal cancer carcinoma; PDAC, pancreatic ductal carcinoma; PC, pancreatic cancer
