# Supplementary material for: Diagnostic Risk Prediction Models for Upper Gastrointestinal Cancers: A Systematic Review
Source: Cancer Epidemiol Biomarkers Prev. 2025 May 22;34(8):1240–51. doi: 10.1158/1055-9965.EPI-24-1714 (PMC12314510; doi:10.1158/1055-9965.EPI-24-1714)
Supplement: Supplementary Figure 1 — shows the risk of bias assessment of included studies [file epi-24-1714_supplementary_figure_1_suppsf1.docx]

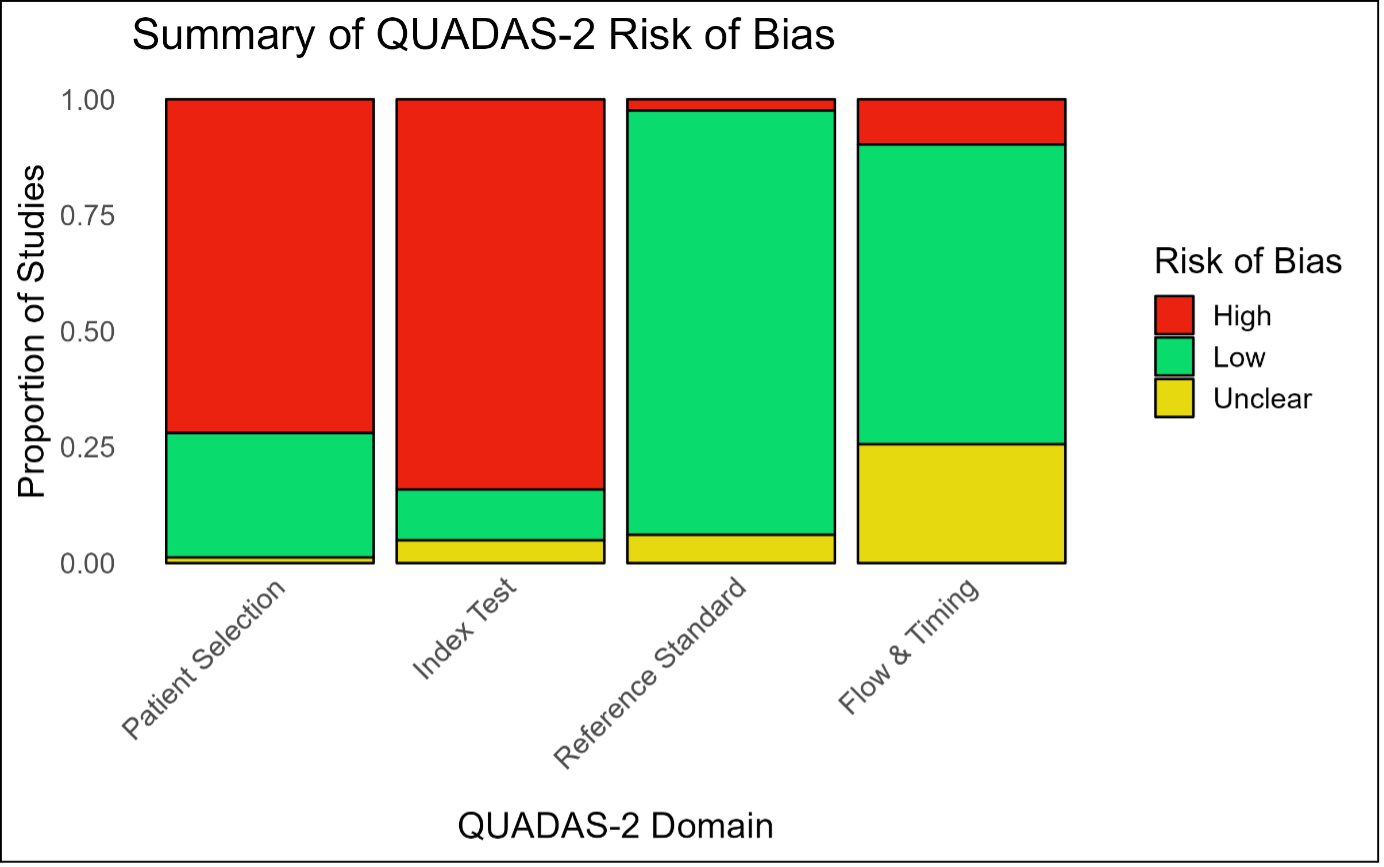


Supplementary Figure 1. Risk of bias assessment using QUADAS-2 framework.

Stacked bar chart showing the proportion of studies rated high (red), low (green) or unclear (yellow) for each domain of the QUADAS-2.
